# The use of incentives in vulnerable populations for a telephone survey: a randomized controlled trial

**DOI:** 10.1186/1756-0500-5-572

**Published:** 2012-10-19

**Authors:** Megan Knoll, Lianne Soller, Moshe Ben-Shoshan, Daniel Harrington, Joey Fragapane, Lawrence Joseph, Sebastien La Vieille, Yvan St-Pierre, Kathi Wilson, Susan Elliott, Ann Clarke

**Affiliations:** 1 Division of Clinical Epidemiology, Department of Medicine, McGill University Health Centre, Montreal, QC, Canada; 2 Division of Pediatric Allergy and Clinical Immunology, Department of Pediatrics, McGill University Health Center, Montreal, QC, Canada; 3 Department of Geography, University of Toronto, Toronto, ON, Canada; 4 Departments of Epidemiology and Biostatistics, McGill University, Montreal, QC, Canada; 5 Food Directorate, Health Canada, Ottawa, ON, Canada; 6 Faculty of Applied Health Sciences, University of Waterloo, Waterloo, ON, Canada; 7 Division of Allergy and Clinical Immunology, Department of Medicine, McGill University Health Center, Montreal, QC, Canada

**Keywords:** Incentives, Vulnerable populations, Response rates

## Abstract

**Background:**

Poor response rates in prevalence surveys can lead to nonresponse bias thereby compromising the validity of prevalence estimates. We conducted a telephone survey of randomly selected households to estimate the prevalence of food allergy in the 10 Canadian provinces between May 2008 and March 2009 (the SCAAALAR study: Surveying Canadians to Assess the Prevalence of Common Food Allergies and Attitudes towards Food LAbeling and Risk). A household response rate of only 34.6% was attained, and those of lower socioeconomic status, lower education and new Canadians were underrepresented. We are now attempting to target these vulnerable populations in the SPAACE study (Surveying the Prevalence of Food Allergy in All Canadian Environments) and are evaluating strategies to increase the response rate. Although the success of incentives to increase response rates has been demonstrated previously, no studies have specifically examined the use of unconditional incentives in these vulnerable populations in a telephone survey. The pilot study will compare response rates between vulnerable Canadian populations receiving and not receiving an incentive.

**Findings:**

Randomly selected households were randomly assigned to receive either a $5 incentive or no incentive. The between group differences in response rates and 95% confidence intervals (CIs) were calculated. The response rates for the incentive and non-incentive groups were 36.1% and 28.7% respectively, yielding a between group difference of 7.4% (−0.7%, 15.6%).

**Conclusion:**

Although the wide CI precludes definitive conclusions, our results suggest that unconditional incentives are effective in vulnerable populations for telephone surveys.

## Background

In 2008/09, we conducted a telephone survey of randomly selected Canadian households to estimate the prevalence of food allergy: the SCAAALAR study (Surveying Canadians to Assess the Prevalence of Common Food Allergies and Attitudes towards Food LAbeling and Risk). A household response rate of only 34.6% was attained, and those of lower socioeconomic status, lower education and new Canadians were underrepresented. The SPAACE study (Surveying the Prevalence of Food Allergy in All Canadian Environments) is attempting to target these vulnerable populations not adequately represented in SCAAALAR.

Poor response rates can potentially lead to nonresponse bias, which can compromise the validity of prevalence estimates. The desired target populations for SPAACE have been found to have lower response rates to surveys and questionnaires; therefore, it is of particular importance for this study to explore methods of maximizing response [1-4]. The positive impact of incentives on response rate has been proven in several mailed questionnaire studies and telephone surveys [1,5-16]. Some studies have specifically examined the use of incentives in low income or high minority populations [1,3,4,17-19]. Unconditional incentives, which are prepaid and not conditional upon participation, have been shown to be more effective than conditional incentives, [9,14,16,20-22] particularly in minority and low income populations [1,3,17-19]. However, no studies have examined the effect of unconditional incentives in Canadian lower socioeconomic status, lower education and immigrant populations in a telephone survey. A telephone survey differs from a mailed questionnaire in that a higher response rate is usually attained [14] and hence, an incentive may not further increase this rate. We conducted a pilot study to evaluate the effect of unconditional incentives on the response rate of vulnerable populations invited to participate in a telephone survey.

## Findings

### Methods

#### Selection of study population

To target the vulnerable populations desired in SPAACE (i.e. those of low socioeconomic status, low education and new Canadians), census tracts from all the census metropolitan areas in the 2006 Canadian census were obtained. Census tracts known to contain either a high proportion of households living under the low income cut-off (income level at which a family has to spend a greater portion of its income on the basics (food, clothing and shelter) than does the average family of similar size) [23] and/or having migrated to Canada since 1996 were identified and converted into postal codes using the 2006 Statistics Canada postal code conversion file. It was thought that there would be considerable overlap between those of low socioeconomic status and those of low education; therefore, non post-secondary graduates were not specifically targeted for sampling. Telephone numbers were randomly selected from these postal codes by Info-Direct, using their database which contains household telephone numbers only associated with names and mailable addresses.

### Administration of incentive

Each household was mailed a letter advising residents that they were going to be telephoned by university affiliated researchers and invited to participate in a 15-minute survey on health and the environment. Letters were addressed to the name associated with each number as it was published in the telephone directory. As we did not want to encourage differential participation among households by food allergy status, the detailed objectives of the study were not disclosed. However, our ethics review board required that we mention that the survey may be more extensive for households with food allergic individuals. To substantiate the legitimacy of the study, the letter informed households that “McGill University” would appear on any caller display technologies for any subject who had a tendency to screen calls. The letter also advised that the study was publicly funded and referred potential participants to a toll free number and a website if they had further queries.

Each of the selected households was randomized to either receive the information letter containing an incentive or the information letter only. The incentive was either a $5 food coupon from a major food manufacturer or a major nationwide coffee shop. Different incentives were used because only a limited supply was donated.

Within two weeks after the mailing of the information letters, trained interviewers, blinded to the presence of the incentive and fluent in both French and English, telephoned each household. Computer Assisted Telephone Interviewing software was used to collect the data. Respondents were considered eligible if they were 18 year or older, were currently living in the household and felt they could answer questions about food allergies and eating habits for all members of the household. If the respondent was not eligible, interviewers attempted to re-schedule a callback at a time when an eligible respondent was available. To optimize response rates, a maximum of 15 callbacks were made at different times and days between 9am and 9pm on weekdays and 10am and 5pm on weekends. Household respondents who refused to complete the survey were asked if they would be willing to complete a shorter questionnaire on food allergy in the household; hereafter referred to as the “abbreviated questionnaire.” In this condensed questionnaire, demographic information was only collected for those households that reported a food allergy.

### Statistical analysis

For both the incentive and non-incentive group, 3 different response proportions were calculated: 1) a response rate, 2) a cooperation rate, and 3) a less conservative cooperation rate. The response rate was defined as the number of completed interviews divided by the number of completed and partial interviews, abbreviated questionnaires, refusals and non-contacts (i.e., households who were never reached). This, however, may be too conservative as it includes all non-contacts as “passive refusals.” A less conservative proportion, the cooperation rate, was calculated by omitting non-contacts from the denominator. The cooperation rate was thus defined as the number of completed interviews divided by the number of completed and partial interviews, abbreviated questionnaires and refusals. A third proportion, the less conservative cooperation rate, was calculated by including the number of partial interviews and abbreviated questionnaires in the nominator and dividing by the number of completed and partial interviews, abbreviated questionnaires and refusals. This proportion is relevant to statistics concerning general prevalence estimates as food allergy prevalence questions were among the first questions asked in the full length questionnaire and were among the limited number of questions asked in the abbreviated version. Thus, it is assumed that the majority of respondents completing a partial interview or an abbreviated questionnaire provided information pertaining to general prevalence estimates.

Demographic characteristics and response, cooperation, and less conservative cooperation rates were compared between groups and 95% confidence intervals (CI) were calculated using a normal approximation to the difference of two binomial distributions.

### Sample size calculations

It was hoped that an approximate gain of 15% in the response rate would be achieved with incentives. Assuming a conservative baseline response rate of 50% and using a total CI width of 20%, it was calculated that a sample size of 184 within both the incentive and non-incentive groups would be required. It was assumed that a significant proportion of these households would be non-contacts. Therefore, the total sample size was nearly doubled to 728 to account for non-contacts.

### Ethics

The research carried out was approved by the Research Ethics Board of the McGill University Health Centre.

## Results

There were no important differences between the incentive and non-incentive group in percentages of households with insufficient fluency in French or English to complete the interview, incorrect telephone numbers, incorrect addresses, or ineligible respondents (Table 1). The proportion of respondents living at or below the low income cut off was higher in the incentive versus non-incentive group (between group difference of 10.8% (95% CI: 0.5%, 21.2%)). The remaining demographic characteristics of respondents did not substantially differ between the incentive and non-incentive groups (Table 2).

**Table 1:** Final dispositions for all households in each group

**Final Disposition**	**Number of Households in Incentive Group (%) n=364**	**Number of Households in Non-Incentive Group (%) n=364**	**Between Group Difference (95% Confidence Interval)**
Completed Interviews	100 (27.5%)	78 (21.4%)	6.0% (−0.2%, 12.3%)
Language Problem ^1^	29 (8.0%)	26 (7.1%)	0.8% (−3.0%, 4.7%)
Partial Interview ^2^	5 (1.4%)	6 (1.6%)	−0.3% (−2.0%, 1.5%)
Problem With Number ^3^	38 (10.4%)	45 (12.4%)	−1.9% (−6.5%, 2.7%)
Non Contact ^4^	53 (14.6%)	59 (16.2%)	−1.6% (−6.9%, 3.6%)
Abbreviated Questionnaire ^5^	26 (7.1%)	27 (7.4%)	−0.3% (−4.0%, 3.5%)
Refusal	93 (25.5%)	102 (28.0%)	−2.5% (−8.9%, 4.0%)
Wrong Address ^6^	17 (4.7%)	18 (4.9%)	−0.3% (−3.4%, 2.8%)
No Qualified Sample ^7^	3 (0.8%)	3 (0.8%)	0.0 (−1.3, 1.3)

^1^ Language problem refers to a respondent who was not fluent in French or English and could therefore not complete the survey.

^2^ Partial interview refers to a respondent who, after beginning the interview, could not finish the entire survey. Partially interviewed subjects all needed to confirm that the given information could be used in research analysis.

^3^ Problem with number refers to numbers which were out of service, fax machine numbers or business telephone numbers.

^4^ Non-Contact refers to respondents who were never reached.

^5^ Abbreviated questionnaire refers to respondents who refused to complete the full interview but who agreed to answer a short series of condensed questions. The abbreviated questionnaire asked if anyone in the household had a food allergy and if the answer was yes, a short series of demographic questions were asked.

^6^ Wrong address refers to letters which were returned due to a wrong address; these households were therefore never contacted.

^7^ No qualified sample refers to households where no eligible participants resided.

**Table 2:** Demographic characteristics for all respondents among participating households stratified by experimental group

	**Individual Respondents in Incentive Group n=255 (for education, n=213)**	**Individual Respondents in Non-Incentive Group n=176 (for education, n=148)**	**Incentive vs. Non-Incentive Between Group Difference (95% Confidence Interval)**
Non-response on income	31.4%	33.0%	−1.6% (−10.6%, 7.4%)
Living at or below the low income cut-off ^1^	33.7%	22.9%	10.8% (0.5%, 21.2%)
Non-response on education (adults only)	11.3%	7.4%	3.8% (−2.2%, 9.8%)
Not a post-secondary graduate ^2^	32.8%	40.1%	−7.3% (−17.9%, 3.2%)
Non-response on birthplace	7.1%	4.0%	3.1% (−1.2%, 7.3%)
Born outside Canada ^3^	46.0%	45.0%	1.0% (−8.8%, 10.8%)

^1^ Individuals living at or below the low income cut-off are representative of those of “lower SES”.

^2^ Non post-secondary graduates are representative of those of “lower education”.

^3^ Individuals born outside Canada are representative of “new Canadians”.

The response rate, cooperation rate and less conservative cooperation rate were all higher in the incentive group (36.1% vs. 28.7%, 44.6% vs. 36.6%, and 58.5% vs. 52.1%, respectively) (Table 3). Response proportions were similar when stratifying by incentive type (Table 4).

**Table 3:** Household response proportions

	**Incentive Group**	**Non-Incentive Group**	**Between Group Difference (95% Confidence Interval)**
Response Rate	36.1% (n=277)	28.7% (n=272)	7.4% (−0.7%, 15.6%)
Cooperation Rate	44.6% (n=224)	36.6% (n=213)	8.2% (−1.6%, 17.7%)
Less Conservative Cooperation Rate	58.5% (n=224)	52.1% (n=213)	6.4% (−3.4%, 16.1%)

n refers to the denominator. The response rate n is larger due to the inclusion of non-contacts.

**Table 4:** Household response proportions stratified by type of incentive

	**Food Coupon #1**	**Food Coupon #2**	**Between Group Difference (95% Confidence Interval)**
Response Rate	36.6% (n=142)	35.6% (n=135)	1.1% (−10.2%, 12.4%)
Cooperation Rate	45.6% (n=114)	43.6% (n=110)	2.0% (−11.0%, 15.0%)
Less Conservative Cooperation Rate	58.8% (n=114)	58.2% (n=110)	0.6% (−12.3%, 13.5%)

n refers to the denominator. The response rate n is larger due to the inclusion of non-contacts.

## Discussion

Canadians living below the low income cut-off, non post-secondary graduates and new Canadians were specifically targeted in this study. These populations are notorious for having low response rates; [1-4,24] therefore, it was particularly important for the SPAACE study to utilise accepted methods of increasing response rates. Hence, randomly selected households were mailed pre-contact letters that were personally addressed and indicated that the researchers were affiliated with Canadian universities and that the study was publicly funded [5]. Potential participants were also referred to a website should they seek further information regarding the study. They were advised that participation was entirely voluntary as it has been shown that stressing personal choice rather than obliging participation results in higher response rates [25]. Further, the letter did not disclose the true purpose of the study, i.e. to assess the prevalence of food allergies, as it was thought that this may introduce selection bias. Households who are actually affected by food allergy may have been more willing to participate, leading to an overrepresentation of those with food allergy and an overestimation of food allergy prevalence [3]. Finally, multiple callbacks were made on difference days and at different times [26].

In addition to the numerous strategies outlined above, we examined the influence of unconditional incentives on the response rate. Based on a predictive model developed by Edwards et al. where the odds of response increased significantly for each $0.01 increase in incentive value up until $5, [9] we chose to use prepaid incentives valued at $5. An increase in response rate, cooperation rate and less conservative cooperation rate of 7.4%, 8.2% and 6.4% respectively were observed in the incentive versus non-incentive group; however, these differences were accompanied by wide CIs, which did not completely exclude very small or even negative values. A larger sample size may have provided smaller CIs making our conclusion more definitive. It was hoped that an increase in response of 15% would be achieved with incentives and although a difference of 15% can be seen in the upper confidence limits of all three response proportions, the width of these CIs makes it unlikely that the true difference is actually this large. Nevertheless, our results suggest that an unconditional incentive likely increases the response rate in our targeted population. Although others have demonstrated the positive effect of incentives on response rates, [6,7,9-11,16,27] ours was the first to examine unconditional incentives in these vulnerable populations for a telephone survey.

Conversely, some studies have reported that incentives may not be beneficial [1,12,28]. Wenemark et al. suggests that the positive impact of incentives may be specific to consumer related research. It is speculated that incentives may insult or annoy potential respondents when the research is health related; that is, subjects may already feel an obligation towards participating in medical research, and offering incentives may induce suspicions of ulterior motives [28].

Some have reported that incentives are more effective among low income or visible minority populations possibly because incentives are of greater value to these individuals [1,3,17-19]. Our results do suggest that incentives may be more effective in lower socioeconomic status populations as a higher proportion of respondents in the incentive group were living at or below the low income cut-off. In contrast, the proportion of respondents without a post-secondary education and born outside Canada was comparable between the groups, suggesting that the incentive was not necessarily more effective in these populations. These conclusions are valid only if we assume that the incentive and non-incentive group had similar demographic characteristics due to the random administration of the incentive.

The ability of incentives to increase participation may be explained by the theory of cognitive dissonance. The theory states that a subject will experience a state of aversive arousal referred to as “dissonance” when they have received an incentive and decide to not participate in the survey. This aversive arousal is a result of the inconsistent idea of receiving something for nothing. To reduce this dissonance, the subject may then decide to participate [25]. Biner and Barton argue that this popular theory contains a flaw, and the more appropriate theory is that of equity. Equity theory states that feelings of guilt result when a subject feels they are being overcompensated for their actions. To reduce this guilt, a subject will choose to participate. The major difference between these theories is that in the equity stream, the potential respondent only considers not participating in the survey whereas in dissonance, the subject makes a decision and subsequently changes their mind [29].

A limitation of our study is that we could not determine if the increased response rate in the incentive group improved the validity of our food allergy prevalence estimates. Although there is a gold standard for determining the prevalence of food allergy, the food challenge, it is potentially very dangerous as the individual is given a food to which they may have a life-threatening allergic reaction. Further, performing a food challenge is very labor intensive, time consuming, and costly and for all these reasons, cannot be applied in this nationwide survey. Therefore, for the purposes of this study, there is no gold standard for determining food allergy. If the prevalence of food allergy did differ between the incentive and non-incentive group, we could not determine which prevalence is more accurate. In addition, the prevalence of allergy to specific foods is at most 2% and therefore our sample size would be too small to accurately estimate any difference between the incentive and non-incentive group. Although it is hoped that a higher response rate will decrease response bias providing a more accurate estimate of food allergy prevalence, it is recognized that the increase in response rate achieved with an incentive does not necessarily automatically decrease response bias. It is possible that only a certain type of non-respondent is encouraged to participate when incentives are utilized, resulting in increased bias, despite the increased response rate.

A second limitation is that the incentive group received two different incentives, one from a major food manufacturer and the other from a nationwide coffee shop. Although both incentives were worth $5, subjects may value these compensations differently; grocery store gift cards can provide necessities whereas coffee shop gift cards cannot. Although it would have been of interest to formally compare the effect of each incentive on the response rate, this would have required a total sample size of over 2000 and was therefore not feasible for a pilot study. Nevertheless, response proportions were calculated when stratified by incentive type (Table 4) and although no differences were observed, a larger sample size may have revealed a meaningful difference. Another limitation of this study was there was no way to confirm that all households in the incentive group actually received the incentive. It was thought that including such a query may influence the interviewers thereby potentially introducing bias. As well, it should be noted that our population was sampled from a directory of telephone numbers associated with mailable addresses and our conclusions may not apply to household telephone numbers without mailable addresses as their response rates may differ.

## Conclusion

When deciding if an incentive is worthwhile, many factors need to be considered, including the importance of increased response, the study budget and timeline. Although wide CIs preclude definitive conclusions, our results suggest that the incremental increase in response rate with unconditional incentives likely merits the additional cost when vulnerable populations are targeted.

## Abbreviations

SCAAALAR: Surveying Canadians to Assess the Prevalence of Common Food Allergies and Attitudes towards Food LAbeling and Risk; SPAACE: Surveying the Prevalence of Food Allergy in All Canadian Environments; CI: Confidence Interval.

## Competing interests

The authors have declared that no competing interests exist.

## Authors’ contributions

All authors contributed to the study design. MK, LS, and JF contributed to the data collection. MK, LJ, and YSP carried out the data analysis. All authors made critical revisions and approved the final manuscript.
